# Biological Functional Relevance of Asymmetric Dimethylarginine (ADMA) in Cardiovascular Disease

**DOI:** 10.3390/ijms141224412

**Published:** 2013-12-16

**Authors:** Sara Franceschelli, Alessio Ferrone, Mirko Pesce, Graziano Riccioni, Lorenza Speranza

**Affiliations:** 1 Department of Medicine and Science of Aging, University G. D’Annunzio-Chieti, Chieti 66100, Italy; E-Mails: s.franceschelli@unich.it (S.F.); alessioferrone@yahoo.it (A.F.); mirkopesce@unich.it (M.P.); 2 Intensive Cardiology Care Unit, San Camillo de Lellis Hospital, San Severo (FG) 71016, Italy; E-Mail: lgriccioni@hotmail.com

**Keywords:** ADMA, nitric oxide, cardiovascular disease

## Abstract

There is growing evidence that increased levels of the endogenous NO synthase inhibitor asymmetric dimethylarginine (ADMA) may contribute to endothelial dysfunction. Studies in animal models as well as in humans have suggested that the increase in ADMA occurs at a time when vascular disease has not yet become clinically evident. ADMA competitively inhibits NO elaboration by displacing l-arginine from NO synthase. In a concentration-dependent manner, it thereby interferes not only with endothelium-dependent, NO-mediated vasodilation, but also with other biological functions exerted by NO. The upshot may be a pro-atherogenic state. Recently, several studies have investigated the effect of various therapeutical interventions on ADMA plasma concentrations.

## 1. ADMA Metabolism

**Figure 1.**
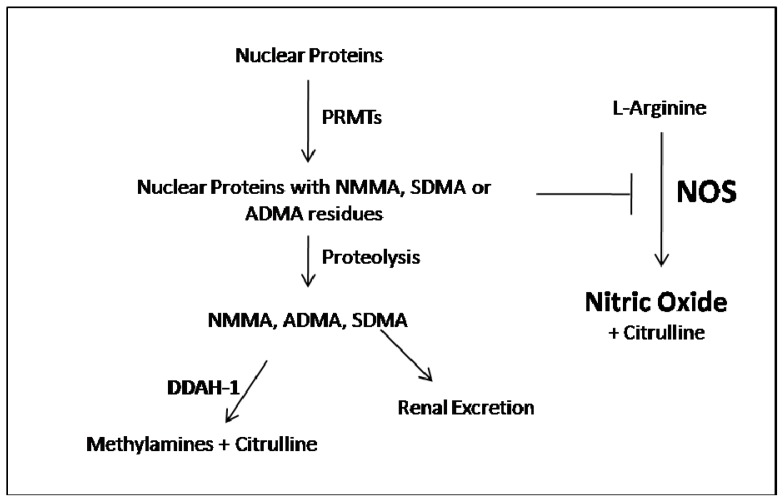
The role of DDAH1 in the metabolism of the nitric oxide synthase (NOS) antagonists asymmetric dimethylarginine (ADMA) and NG-monomethylarginine (NMMA). PRMTs, protein arginine methyltransferases; SDMA, symmetrical dimethylarginine.

Asymmetric dimethylarginine (ADMA) and NG-monomethylarginine (NMMA) are endogenous guanidine-substituted analogs of l-Arginine (l-Arg) that inhibit *in vivo* nitric oxide (NO) synthesis by competing with l-Arg at the active site of NO synthase. Nitric oxide is a potent vasodilator that plays a critical role in maintaining vascular homeostasis through its anti-atherogenic and anti-proliferative effects on the vascular wall and its altered biosynthesis has been implicated in the pathogenesis of cardiovascular disease [1,2]. In addition to blocking NO formation, NMMA and ADMA can uncouple NO synthase leading to the generation of superoxide [3,4]. ADMA is synthesized by proteolysis of post-translationally methylated arginine in proteins [5]. This post-translation modification is done by enzymes called protein-arginine methyltransferases (PRMTs) [6]. In mammalian cells, these enzymes have been classified as type I (PRMT 1, 3, 4, 6, and 8) and type II (PRMT 5, 7, and FBXO11): in the first reaction both enzymes catalyze the formation of NMMA from l-Arg, but in the second reaction, type I PRMTs produces ADMA, while type II PRMT produces symmetric dimethylarginine (SDMA). During protein turnover, methylarginines are released in the cytoplasm. While SDMA is eliminated almost entirely by renal excretion, ADMA is extensively metabolized intracellularly through the activity of enzymes called dimethylarginine dimethylamino hydrolase (DDAH) to citrulline and dimethylamine (DMA), although some ADMA is also excreted by the kidney (Figure 1) [7].

The two identified isoforms of DDAH are encoded by genes located on chromosomes 1 (DDAH-1) and 6 (DDAH-2) and have distinct tissue distributions. DDAH-1 is the predominant isoform in the proximal tubules of the kidney and in the liver. It has been estimated that more than 70% of ADMA is metabolized in these organs which extract ADMA from the circulation. DDAH-2 is the predominant isoform in the vasculature, where it is found in endothelial cells adjacent to the cell membrane as well as in intracellular vesicles and in vascular smooth muscle cells among the myofibrils and the nuclear envelope. In the kidney and liver, a newly recognized elimination pathway for ADMA is transamination to α-keto-d-(N(G),N(G)-dimethylguanidino) valeric acid (DMGV) by the enzyme alanine-glyoxylate aminotransferase 2 (AGXT2) [8]. ADMA is both exported from its site of origin and imported from the plasma at distant sites by cationic aminoacid transporters (CATs) in exchange for arginine and other cationic amino acids. CATs are widely distributed on cell membranes either as high-affinity, low-capacity transporters, like CAT-1, which transport ADMA and arginine across cell membranes in blood vessels and the distal nephron of the kidney, or as higher-capacity, lower-affinity transporters, like CAT-2A, which transport these cationic aminoacids across the membranes of liver cells [9]. In most studies, plasma levels of ADMA in humans and rats are in the range of 0.3 to 0.5 μmol/L. Estimates of intracellular ADMA concentrations suggest that ADMA levels in cells are 10 or 20 times higher than in plasma. Erythrocytes play an important role in the storage and generation of endogenous NOS inhibitor [10]. There is fast bidirectional traffic of ADMA across the plasma membrane of the erythrocyte, leading to equilibrium between intra- and extracellular ADMA. Upon lysis of erythrocytes, proteolytic activity leads to a substantial release of free ADMA from methylated proteins [11].

## 2. ADMA in Oxidative Stress, Inflammation and Cardiovascular Disease

Nitric oxide is a free radical produced in mammalian cells constitutively or induced by various cell activators through the oxidation of l-arginine by a family of iso-enzymes known as nitric oxide synthase (NOS): nNOS (neuronal), eNOS (endothelial) and iNOS (inducible) [12,13]. ADMA is an endogenous inhibitor of all three isoforms of NOS. *In vitro* and *in vivo*, it has been proved that ADMA can compete with the l-arginine substrate, reducing NO formation, a molecule of particular interest in cardiovascular disease [14,15].

There is indeed strong evidence that NO plays a critical role in pre-atherogenic endothelial dysfunction [16]. Although now undergoing a degree of reappraisal, direct targeting of the NO pathway has proved to be of limited use to date. NO donors can experience substantial adverse effects such as headache along with potential production of free radicals and the development of nitrate tolerance, the precise mechanisms of which remain unclear [17,18]. Conversely, direct inhibition of NO synthesis in vasodilatory septic shock, a condition of pathological NO excess, has shown no survival benefit in randomized trials [19]. Targeting regulators of the NO pathway rather than increasing NO directly has proved to be a successful approach as illustrated by the use of phosphodiesterase-5 inhibitors in pulmonary hypertension and erectile dysfunction [20]. This strategy might allow both tissue-specific targeting of pharmacological actions and potential enhancement or inhibition of endogenous homeostatic mechanisms, e.g., acting only where NO synthesis is dysregulated while preserving constitutive NO production. Furthermore, there is evidence that a number of vascular conditions are characterized by aberrant regulation of NO synthesis, suggesting that some of these endogenous control mechanisms might not only provide potential therapeutic targets but also underlie the pathogenesis of chronic cardiovascular diseases [21]. The endogenous methylarginines are candidates as NO pathway regulators in the above respects. There is not only evidence that these molecules modulate NO *synthesis in vivo*, but also that this pathway has a primary role in the pathogenesis of disease. In addition, a degree of tissue/cell specificity of the enzymes controlling methylarginine levels, along with potential for playing a key homeostatic role in NO synthesis, makes this pathway an attractive target for therapeutic intervention [22,23]. There have been a series of *in vitro*, *in vivo*, and clinical investigations designed to characterize the pathological role of the endogenous methylarginines in human health and disease (Table 1).

**Figure 2.**
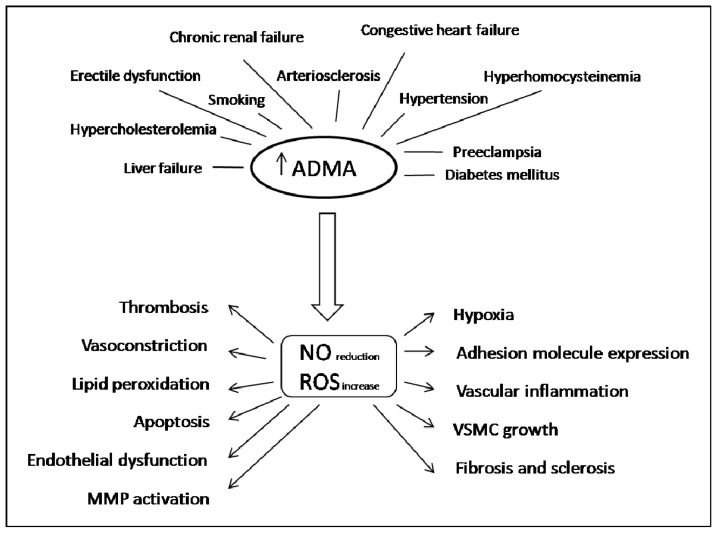
Flow diagram outlining clinical conditions that have been reported to be associated with elevated ADMA concentration, and the interactive roles of ROS and ADMA in relating cardiovascular risk factors to pathophysiological changes in tissues that may underlie cardiovascular disease. ROS, reactive oxygen species; MMP, matrix metalloproteinase; VSMC, vascular smooth muscle cell.

Specifically, the role of ADMA has been the focus of interest in cardiovascular conditions [34,35]. However, the accumulating evidence suggests that a simplistic strategy targeting circulating ADMA in chronic disease may not be successful. This editorial reviews the biology, summarizes the state of the field, details some of the controversies, and proposes avenues for further study. ADMA has been suggested as a novel independent risk factor for endothelial dysfunction and coronary heart disease. Increased plasma levels of ADMA have been documented in several conditions that are characterized by endothelial dysfunction, including hypertension, hypercholesterolemia, hyperglycemia, renal failure and tobacco exposure (Figure 2) [22,29,36].

Plasma ADMA levels are increased in Peripheral arterial disease (PAD) compared to age- and gender-matched controls, and are associated with a reduction in urinary nitrogen oxides [37].

Finally, increased ADMA serum levels are involved in the development of pulmonary vascular disease in scleroderma subjects with pulmonary artery hypertension (PAH) [38].

There is a strong association between oxidative stress, stimulation of PRMT and inhibition of DDAH, increased endothelial ADMA concentrations and eNOS-mediated superoxide production [25]. It is still unclear whether increased ROS production is responsible for increased ADMA levels or whether increased production of ADMA contributes to oxidative stress. In endothelial cells, during a state of oxidative stress such as is observed after ischemia reperfusion [30] or is induced by high glucose medium [39], there is a reduction of DDAH expression. Increased ADMA concentrations in cultured endothelial cells or in patients with endothelial dysfunction are associated with increased reactive oxygen species (ROS) production via regulation of the downstream NO signaling pathway [35,40,41]. Several findings suggest that ADMA may contribute to oxidative stress by causing eNOS uncoupling via depletion of tetrahydrobiopterin (BH4) resulting in a shift from NO production to superoxide production. The PRMT and DDAH activity is redox-sensitive [42,43]. Accumulating evidence has documented that ADMA may induce a vascular inflammation reaction prompting the development of cardiovascular disease by activation of leukocyte adhesion and cytokine production [44]. So ADMA is not only a risk factor for endothelial dysfunction but also a novel pro-inflammatory mediator. Plasma ADMA levels arereported to be elevated in patients with chronic HF, and to correlate significantly with New York Heart Association (NYHA) functional class and exercise capacity [45].

In conclusion, ADMA is not only a marker but also a mediator of oxidative stress. Oxidative stress and inflammation play a pivotal role in ADMA pathophysiology by managing PRMT/DDAH expression and NO synthesis and leading to endothelial dysfunction. Thus, ADMA acts as a linker between endothelial dysfunction and inflammation and plays a crucial role in cardiovascular morbidity and mortality.

## Figures and Tables

**Table 1.** Overview of the *in vitro*, *animal* and *human* studies on ADMA.

	Model	ADMA results	Final result	Author
***In vitro***	INS-1 cells BEAS-2B cells	In INS-1 cells: (1) ADMA (0.05–32 μM) increased insulin release *in vitro*, except at a high concentration (32 μM); (2) ADMA stimulated the production of IL-6 and MIP-2. In BEAS-2B cells ADMA did not cause any increase in IL-8 or TNF-α or RANTES secretion.	ADMA has a pathophysiological impact leading to a diabetic situation but has no impact on the respiratory system.	[24]
	Cultured primary human vascular endothelial cells (ECs)	PD 404182 significantly increased intracellular levels of ADMA.	PD 404182 directly and dose-dependently inhibits DDAH and reduced lipopolysaccharide (LPS)-induced NO production.	[25]
**Animal**	Mouse model of Polycystic ovary syndrome (PCOS)	DHT (dihydrotestosterone) treatment (compared with placebo) induced no change in plasma ADMA levels.	In DHT-exposed mice, hyperandrogenemia specifically decreases endothelium dependent vasorelaxation without deterioration of smooth muscle function.	[26]
	Male Sprague–Dawley rats	Chronic endogenous infusion of ADMA leads to a significant elevation of plasma ADMA levels.	Chronic elevated plasma levels of ADMA in healthy rats did not affect the number of peripheral blood cells including leukocyte subset.	[27]
	Dahl salt-sensitive (SS/JrHsd)	Serum ADMA and NO concentration remained unchanged between the baseline and 6-weeks study period in the HS-fed animals despite the treatment group. Both ADMA and NO levels were not favorably affected by treatment with INT-747.	High-salt diet downregulated DDAH expression while treatment with INT-747 protected the loss in DDAH expression and enhanced insulin sensitivity compared to vehicle controls.	[28]
	db/db mice	Silibinin administration markedly decreased plasma ADMA; consistently, aorta ADMA was reduced in silibinin-treated animals.	Silibinin markedly improves endothelial dysfunction in db/db mice by reducing circulating and vascular ADMA levels.	[29]
	Rats	Both plasma and renal concentrations of ADMA increased after renal ischemia followed by reperfusion.	Ischemia-reperfusion injury leads to reduced DDAH activity and modification of different DDAH isoform expression, thus leading to increased ADMA levels, which may lead to increased cardiovascular risk.	[30]
**Human study**	Acute congestive heart failure	ADMA and SDMA plasma levels were significantly higher after pharmacological treatment respect to baseline values.	In patients with ACHF, acute renal impairment function and the modulation of metabolism and extracellular transport by the DDAH-1/CAT-1 system determine high ADMA and SDMA levels after therapy for acute congestive heart failure.	[31]
	Healthy humans	ADMA, SDMA, MMA and arginine levels were significantly higher in PBMC than in plasma, whereas homoarginine levels were not significantly different.	In healthy individuals, plasma levels of arginine, MMA, ADMA, and SDMA poorly reflect their intracellular levels in PBMC.	[32]
	Peripartum cardiomyopathy (PPCM)	ADMA was significantly higher in serum from PPCM patients compared to healthy postpartum women.	Increased levels of Cathepsin D activity, miR-146a and ADMA in serum of PPCM patients support the pathophysiological role of 16 kDa Prolactin for PPCM and may be used as a specific diagnostic marker profile.	[33]
